# Role of TDP‐43 in dysregulation of cholesterol metabolism and implications for AD

**DOI:** 10.1002/alz.093221

**Published:** 2025-01-03

**Authors:** Khushbu Kabra, Elizabeth M Bradshaw

**Affiliations:** ^1^ Columbia University, NEW YORK, NY USA; ^2^ Department of Neurology, Columbia University, New York, NY USA; ^3^ Taub Institute for Research on Alzheimer’s Disease and the Aging Brain, Columbia University, New York, NY USA

## Abstract

**Background:**

Lipid dysregulation is a known feature of Alzheimer’s Disease. Importantly, alterations in lipids pathways affect immune responses in cells like microglia, which have been shown to accumulate cholesterol in both aging and neurodegeneration. Recently, the presence of TDP‐43 inclusions has been linked to increased severity of cognitive impairment in AD patients. While TDP‐43 inclusions are associated primarily with ALS, their presence has been observed in around 57% AD patients. The link between TDP‐43, cholesterol dyshomeostasis and defects in immune responses in the context of AD is yet to be uncovered.

**Method:**

**Microglia**: Peripheral blood mononuclear cells (PBMCs) are isolated and microbead separation is used to further purify monocytes. These monocytes are cultured with a cytokine cocktail for 10‐14 days to induce differentiation into MDMi (monocyte derived microglia). N = 17 **Knockdown screen**: Lentivirus mediated shRNA knockdown is used to reduce expression of various genes implicated in AD and ALS, including TREM2, CD33 and TARDBP in MDMi. **Functional assays**: Cholesterol uptake is measured by using a fluorescence microplate reader and lipid droplets are stained using LipidTox. Gene expression is measured using Fluidigm.

**Result:**

TARDBP knockdown resulted in the most significant downregulation of several cholesterol metabolism associated genes, including ABCG1, NPC1, CYP27A1, LXR and SREBP1/2, which are involved in cholesterol uptake, storage and synthesis. The TARDBP knockdown MDMi also had reduced cholesterol uptake and lowered response to LPS stimulation, compared to other gene knockdowns.

**Conclusion:**

TARDBP plays an important role in the regulation of cholesterol metabolism, specifically cholesterol uptake. The presence of TDP‐43 inclusions in AD may thus contribute to AD pathology through loss of function of TARDBP, causing defects in cholesterol homeostasis which lead to altered microglia function. This also has implications in better understanding Limbic‐predominant age‐related TDP‐43 encephalopathy (LATE), a recently characterized type of dementia associated with TDP‐43 proteinopathy.

